# Strategies for delivering drugs across the blood-brain barrier for the treatment of neurodegenerative diseases

**DOI:** 10.3389/fddev.2025.1644633

**Published:** 2025-08-26

**Authors:** Xiaopei Zhang, Manfei Fu, Yuanfei Wang, Tong Wu

**Affiliations:** ^1^ Shandong Key Laboratory of Medical and Health Textile Materials, Collaborative Innovation Center for Eco-textiles of Shandong Province and the Ministry of Education, College of Textiles and Clothing, Qingdao University, Qingdao, China; ^2^ Medical Research Center, The Affiliated Hospital of Qingdao University, Qingdao Medical College, Qingdao University, Qingdao, China; ^3^ Qingdao Stomatological Hospital Affiliated to Qingdao University, Qingdao, China

**Keywords:** blood-brain barrier, drug delivery, neurodegenerative diseases, nanocarriers, targeted delivery

## Abstract

The blood-brain barrier (BBB) restricts development of drug delivery systems for brain, which hinders the potential applications of numerous pharmaceutical agents for treating central nervous system (CNS) diseases. A number of drug delivery systems have been developed to enhance the capacity of drugs to cross BBB. A detailed introduction of the structure and function of BBB was given based on the mechanism of BBB, while comparing with the pathological changes of BBB in neurodegenerative diseases (NDDs), including activation of endothelial cells, the loose of tight junction and increase of BBB permeability. The liposomes, polymer nanoparticles and other novelty approaches for treating NDDs were summarized. Here, we provide a novel perspective to classify the strategies of drug delivery system as passive targeting and active targeting according to their mechanisms. The potential of clinical translational for drug delivery systems in NDDs was explored and underscored the imperative of safety and verification through clinical trials. In summary, this review proposed current developments of drug delivery systems and discussing the potentials of drug delivery systems in clinical translational which bring new breakthroughs for treating NDDs.

## 1 Introduction

Neurodegenerative diseases (NDDs) are a group of chronic progressive conditions characterized by the gradual deterioration for the structure and function of neural cells in the central nervous system (CNS) (Wilson et al., 2023). These diseases have complex pathological mechanisms involving the gradual loss of neurons, imbalances in neurotransmitters, and neuroinflammation, among other factors (Dileep et al., 2023). They primarily include Alzheimer’s disease (AD) (Miller et al., 2022), Parkinson’s disease (PD) (Gan et al., 2025), amyotrophic lateral sclerosis (Wilkins et al., 2024), and Huntington’s disease (Aviner et al., 2024). Among these, AD is the most common NDDs, characterized by memory impairment, cognitive decline, and behavioral changes, significantly impacting patients’ quality of life (Jack, 2022). PD primarily manifests as motor dysfunction, such as tremors, muscle rigidity, and bradykinesia, causing significant inconvenience to patients’ daily lives (Paul et al., 2023). These diseases not only impose heavy economic and psychological burdens on patients and their families but also place enormous strain on societal healthcare resources. The incidence of NDDs worldwide is on the rise, with projections indicating that by 2050, the number of AD patients and PD patients will also increase significantly (Su et al., 2025; Nichols et al., 2019; Ahmadi-Abhari et al., 2017). Therefore, in-depth research into the pathogenesis of NDDs and the development of effective treatment approaches have become a global hotspot and challenge in medical research.

The blood-brain barrier (BBB) is an important physiological barrier of the CNS, composed of cerebral capillary endothelial cells, tight junctions, basement membranes and pericytes. Its main function is to maintain the stability of the internal environment of CNS, preventing harmful substances from entering the brain, and regulating the transportation of nutrients (Ayloo and Gu, 2019; Banks, 2016). The presence of BBB is crucial for protecting the brain from external toxins and pathogens, but it also as a challenge for drug delivery to treat NDDs. More than 98% of small-molecule drugs and nearly 100% of large-molecule drugs (e.g., proteins, antibodies, and gene therapy drugs) exhibit low efficiency penetrating the BBB, resulting in the drug concentration in the brain being much lower than the therapeutic level (Pandit et al., 2020a; Banks, 2016). In addition, patients with NDDs often experience BBB dysfunction, such as disrupted tight junctions (Shi et al., 2025) and intensified inflammatory responses (Wei et al., 2024), which further limit the efficiency of drug delivery. The change of the BBB permeability is closely related to the progress of NDDs. However, there are currently no established solutions for effectively enhancing drug penetration through the BBB.

Traditional drug delivery methods, such as systemic administration, often require high doses to achieve effective concentrations in the brain, which not only increases the drug’s toxic side effects but also limits its clinical application. Hence, developing novel drug delivery strategies to enhance drug BBB penetration capacity and brain distribution efficiency has become a key breakthrough in the treatment of NDDs. In recent years, with the interdisciplinary development of nanotechnology, biotechnology, and other fields, various innovative drug delivery strategies have emerged, bringing new hope for the treatments of NDDs (Tang et al., 2025; Ji et al., 2024; Wang H. et al., 2025). These strategies not only enhance drug BBB penetration but also offer advantages such as target delivery and good biocompatibility, potentially playing a significant role in future clinical treatments. However, these strategies are still not available for clinical treatments and face numerous challenges, such as the stability of drug delivery systems, long-term safety, and the feasibility of clinical translation. Thus, conducting in-depth researches into the mechanisms of action of these strategies, optimizing their design and preparation processes, and validating their safety and efficacy through clinical trials are currently important directions in the research of neurodegenerative disease treatment. This review provides a systematic overview of the latest research advances in drug delivery strategies that penetrate the BBB, conducting an in-depth analysis of the mechanisms, advantages, and challenges of various strategies, and exploring their potential applications in the treatment of NDDs. The aim is to provide a reference for related research and to promote the development of this field.

## 2 Blood-brain barrier

### 2.1 Structure and function of BBB

**FIGURE 1:**
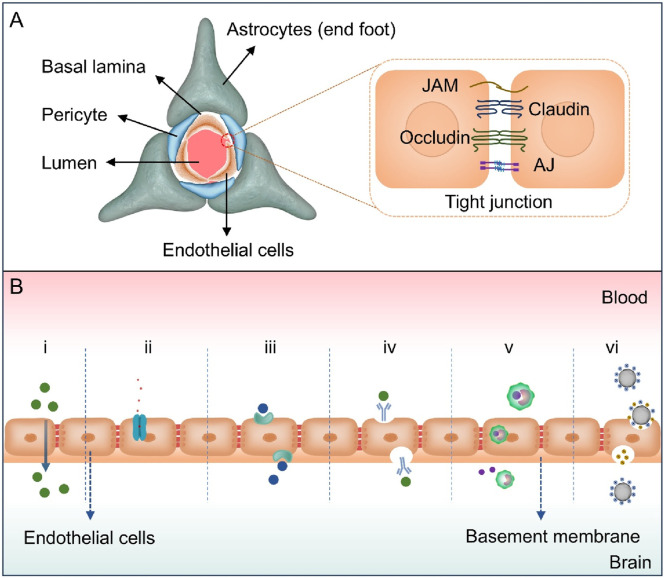
**(A)** The structure of the BBB. The BBB is mainly composed of brain microvascular endothelial cells, pericytes, astrocyte end feet, and basement membranes. Brain microvascular endothelial cells form a continuous barrier structure through tight junctions, which are composed of proteins such as Claudin, Occludin, junctional adhesion molecule (JAM) and adhesion junction (AJ), and restrict the free permeability of substances. **(B)** Diagram illustrating various mechanisms for crossing the BBB, including (i) passive diffusion (ii) efflux pumps, (iii) carrier-mediated transcytosis, (iv) receptor-mediated transcytosis, (v) cell-mediated transcytosis, and (vi) adsorptive-mediated transcytosis.

BBB plays a crucial role in preserving the internal environment of CNS (Shi et al., 2025; Upton et al., 2022). Its selective barrier function is contingent on the collaborative effects of brain microvascular endothelial cells, a basement membrane, pericytes, and astrocytes (Figure 1A; Pandit et al., 2020a). Among them, brain microvascular endothelial cells serve as the core functional units of BBB (Langen et al., 2019). They form the basis of a physical barrier through tight junctions constructed by proteins such as claudins and occludins, directly restricting the free diffusion of polar molecules and macromolecular substances. In the meanwhile, they achieve the uptake of essential nutrients and clearance of exogenous substances via carrier proteins (e.g., glucose transporter, GLUT) and efflux pumps (e.g., P-glycoprotein, P-gP) on the cell membrane, realizing regulation of transmembrane transport processes. The basement membrane, as a structural support for endothelial cells, is composed of components such as collagen and laminin. It is responsible for maintaining the mechanical integrity of the vascular wall, regulating barrier stability through dynamic interactions with endothelial cells and pericytes, and providing a medium for intercellular signal transmission. Pericytes are located on the outer side of endothelial cells and secrete signaling factors, such as vascular endothelial growth factor and transforming growth factor-beta. These factors are important in the dynamic regulation of endothelial tight junction integrity and permeability. Furthermore, pericytes are involved in the processes of angiogenesis and vascular maturation (Rustenhoven et al., 2017). Astrocytes’ end feet, which extensively cover the surface of blood vessels, promote endothelial cell differentiation and enhance tight junction stability by releasing growth factors such as brain derived neurotrophic factor. They also indirectly regulate nutrient transport efficiency by sensing metabolic demands in the brain, further strengthening barrier function (Zhao et al., 2015).

The structural characteristics of BBB give it the ability to prevent harmful substances from invading the brain and allow essential nutrients to pass through. Due to the compact nature of BBB, paracellular transportation of molecules is significantly restricted. The predominant mechanisms of drug and essential molecule entry into the brain include passive diffusion, efflux pumps, carrier-mediated transport, receptor-mediated transcytosis (RMT), cell-mediated transcytosis and adsorptive-mediated transcytosis (AMT, Figure 1B) (Liu et al., 2025). Passive diffusion is defined as the movement of lipophilic small molecules (e.g., alcohol, steroid hormones, dexamethasone) along a concentration gradient, devoid of energy, and exhibiting non-saturability. The efficiency of the process is associated with various factors, including molecular weight (<500 Da), lipophilicity (LogP>2), the number of hydrogen bonds formed (<6), and polar surface area (PSA<60 to 70 square angstroms) (Hladky and Barrand, 2018; Zha et al., 2024). Paracellular diffusion is restricted by tight junctions, while transcellular diffusion depends on molecular permeability. Adenosine triphosphate (ATP)-binding cassette superfamily efflux transporters, including P-gp and multidrug resistance-associated protein family, expressed by BBB endothelial cells which facilitate the expulsion of drugs from these cells, leading to impeding their entry into the brain (Lopez-Mitjavila et al., 2025; Balzer et al., 2022). These transporters are localized to the basolateral and luminal sides of the cell membrane based on substrate differences.

Carrier-mediated transcellular transport is a process that utilizes highly selective transporters (gucose transporter 1 and L-type amino acid transporter 1, LAT1) to facilitate the movement of nutrients such as glucose and amino acids, as well as drugs with structural similarities (Zhou et al., 2020; Arora et al., 2020). A subset of these processes is contingent upon ATP for the purpose of energy supply and is executed in an inverse concentration gradient. RMT relies on specific receptors on the lumen side of cerebral capillary endothelial cells (Zhang et al., 2020). These receptors include insulin receptors, transferrin receptors, and so on (Fan et al., 2018). The selective uptake of specific macromolecules was improved through receptor reuptake mechanisms RMT, vesicle transport mechanisms and a series of ligand-receptor binding events. AMT is initiated by electrostatic interactions between positive charges of the macromolecule and the anionic components of endothelial cell membrane. The transportation of polycationic proteins, cell-penetrating peptides, and related molecules occurs through this pathway (Muniswamy et al., 2019; Oka et al., 2023). Cell-mediated transcytosis is a pathway that has been discovered in recent years. A recent study has devised a hybrid system of “Trojan horse”-like nanocapsules conjugated with Th17 cells, which can be injected intravenously and cross the BBB to target the inflammatory lesions of multiple sclerosis (MS). Under the stimulation of reactive oxygen species (ROS), it releases trans-differentiation inducers to induce the *in situ* trans-differentiation of Th17 cells into Treg cells for the treatment of MS (Shi et al., 2023). This mechanism has been investigated and implemented in the domain of drug delivery for inflammation-related neurological diseases.

### 2.2 Pathological changes of BBB in NDDs

**FIGURE 2:**
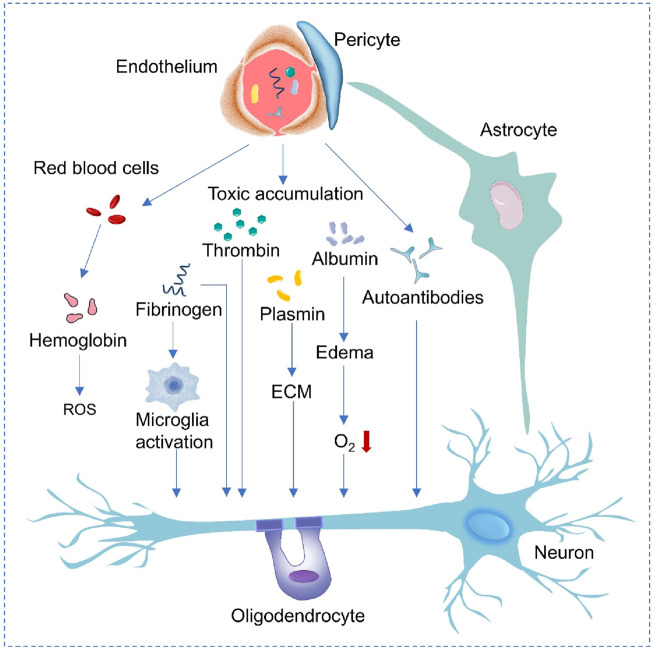
Mechanisms of neurodegeneration associated with blood vessels. When the endothelial cells and pericytes of the BBB are damaged, red blood cells leak and release hemoglobin, generating ROS. Fibrinogen leakage activates microglia. Barrier dysfunction leads to the accumulation of thrombin, albumin and autoantibodies, causing plasmin activation, changes in extracellular matrix (ECM) and edema. The above-mentioned events finally damage the oxygen (O_2_) supply to neurons, affect the functions of astrocytes, oligodendrocytes and neurons, and promote the progression of neurological diseases.

Disruptions in the BBB typically occur in conjunction with and contribute to the progression of NDDs, and various vascular problems are often closely linked to neurodegenerative changes (Figure 2; Zhao et al., 2015; Omar et al., 2025). In AD, when the function of the BBB is impaired, neurotoxic proteins such as beta-amyloid peptide (Aβ) and Tau protein cannot be effectively cleared and will accumulate in the cerebral blood vessels and brain parenchyma, accelerating neuronal damage and neurodegeneration (Lee et al., 2022; Feng et al., 2020; Kaji et al., 2024). The process can be improved by targeting RMT strategies. Low-density lipoprotein receptor-associated protein 1 (LRP1) and other receptors on the surface of BBB endothelial cells facilitate delivery of nanocarriers carrying antibodies to the brain through transendocytosis to enhance clearance of toxic proteins. Downregulation of LRP1 in the damaged BBB may necessitate pretreatment with drugs to restore function and delivery efficiency. Similarly, abnormal aggregated proteins such as α-synuclein (α-SYN) cannot be cleared or transported in time due to abnormal BBB function, and then deposit in the brain, exerting toxic effects on nerve cells and promoting the progression of the PD (García-Revilla et al., 2023; Wang H. et al., 2025; Chu et al., 2024). Dysregulation of the inflammatory response is also an important feature of NDDs (Zhang W. et al., 2023). BBB endothelial cells can be activated, leading to increased anionic components (like heparin proteoglycans) on their surfaces. These changes can be used to create multi-cationic delivery vehicles (like nanoparticles modified with cationic peptides), which can deliver AMT-mediated α-SYN scavengers or anti-inflammatory drugs via electrostatic interactions. However, the activation of endothelial cells may also lead to less-specific distribution of the delivery vector (Arora et al., 2024).

The opening of the BBB occurs in the early stage of the MS, and peripheral immune activation precedes pathological immune activation in the CNS. The CNS is entered by immune cells, such as lymphocytes, through the damaged BBB, and inflammatory mediators, such as cytokines, are released. An inflammatory response is triggered, and further damage is caused to neurons and myelin sheaths (Zierfuss et al., 2024; Hermans et al., 2022; Qu et al., 2024). The infiltration of peripheral immune cells and inflammatory mediators into the brain tissue is caused by the destruction of BBB, which intensifies the neuroinflammatory response. And then, the inflammation makes the BBB worse, and it is like a vicious cycle where the neuronal damage gets worse and worse (Chou et al., 2023; Heneka et al., 2025; Wang S. et al., 2024; Schneeberger et al., 2025; Chu et al., 2024). Research has demonstrated that α-SYN stimulates microglia through the engagement of multiple pattern recognition receptors, including Toll-like and Fcγ receptors. When α-SYN aggregation is recognized by these receptors, the NF-κB pathway is activated, which leads to the release of pro-inflammatory cytokines, promoting further neuroinflammation (Wang et al., 2023). During this process, molecules like intercellular cell adhesion molecule-1 and vascular cell adhesion molecule-1 increase on inflamed endothelial cells’ surfaces, and can be taken in by cell-mediated transocytosis (Wang et al., 2022). Endothelial cells’ tight junctions may also be loosened, increasing the exposure of transporters like LAT1 in vector-mediated transcellular transport. Drug molecules designed to resemble amino acids linked to inflammation can enhance delivery through LAT1 (Sun et al., 2022; Shi and Yong, 2025). Inflamed endothelial cells may also have changes in the expression or distribution of receptors, e.g., insulin receptors, which can affect RMT. Therefore, the selection of targeted ligands needs to be adjusted according to the changes in receptors in the inflammatory environment (Omar et al., 2025; Varatharaj and Galea, 2017; Wei et al., 2024).

The enhanced state of oxidative stress, coupled with the inadequate regulatory impact of BBBs on oxidative stress, allows harmful free radicals and oxidative stress factors to penetrate the brain more easily (Plascencia-Villa and Perry, 2023). This results in increased oxidative damage to neurons, impaired neuronal function, and the progression of the disease (Dash et al., 2025; Liu et al., 2024). Oxidative stress products, which include reactive oxygen species resulting from mitochondrial dysfunction, can accumulate in the brain and potentially damage nerve cells. The accumulation has the potential to influence the blocking and clearance functions of the brain barrier, and can interfere with the synthesis, release, and reuptake of neurotransmitters, resulting in abnormal neurological functions (Xi et al., 2025; Song N. et al., 2025). Abnormal cerebral circulation is also an important pathological feature of NDDs (Sweeney et al., 2018). In addition, impaired function of vascular endothelial cells can lead to increased vascular permeability and abnormal vasoconstriction function, affecting the blood supply to the brain and aggravating nerve damage (Feng et al., 2025). Recent studies have shown that during aging and the occurrence of NDDs, the glycocalyx layer on the surface of brain endothelial cells becomes disordered, especially the abnormal glycoproteins in the mucin domain, which can lead to a decline in barrier function and even induce cerebral hemorrhage, making it easier for neurotoxic substances and inflammatory factors to enter the brain and exacerbating nerve damage (Shi et al., 2025). Briefly, the BBB can be damaged by various factors, which disrupts permeability and complicates drug delivery control. Meanwhile, abnormal immune responses reduce its effectiveness, making the treatment of NDDs difficult.

## 3 Types and applications of drug delivery systems for the BBB

The treatment of CNS diseases has been hindered by the BBB. In order to effectively overcome this obstacle, scientists have developed a variety of advanced materials and delivery systems in recent years. The objective of these developments is to enhance the targeting and delivery efficiency of drugs in the brain and to achieve effective cross-BBB drug delivery. This section will provide a detailed introduction to the types of major drug delivery systems and their applications in the treatment of CNS diseases.

### 3.1 Liposomes

**FIGURE 3:**
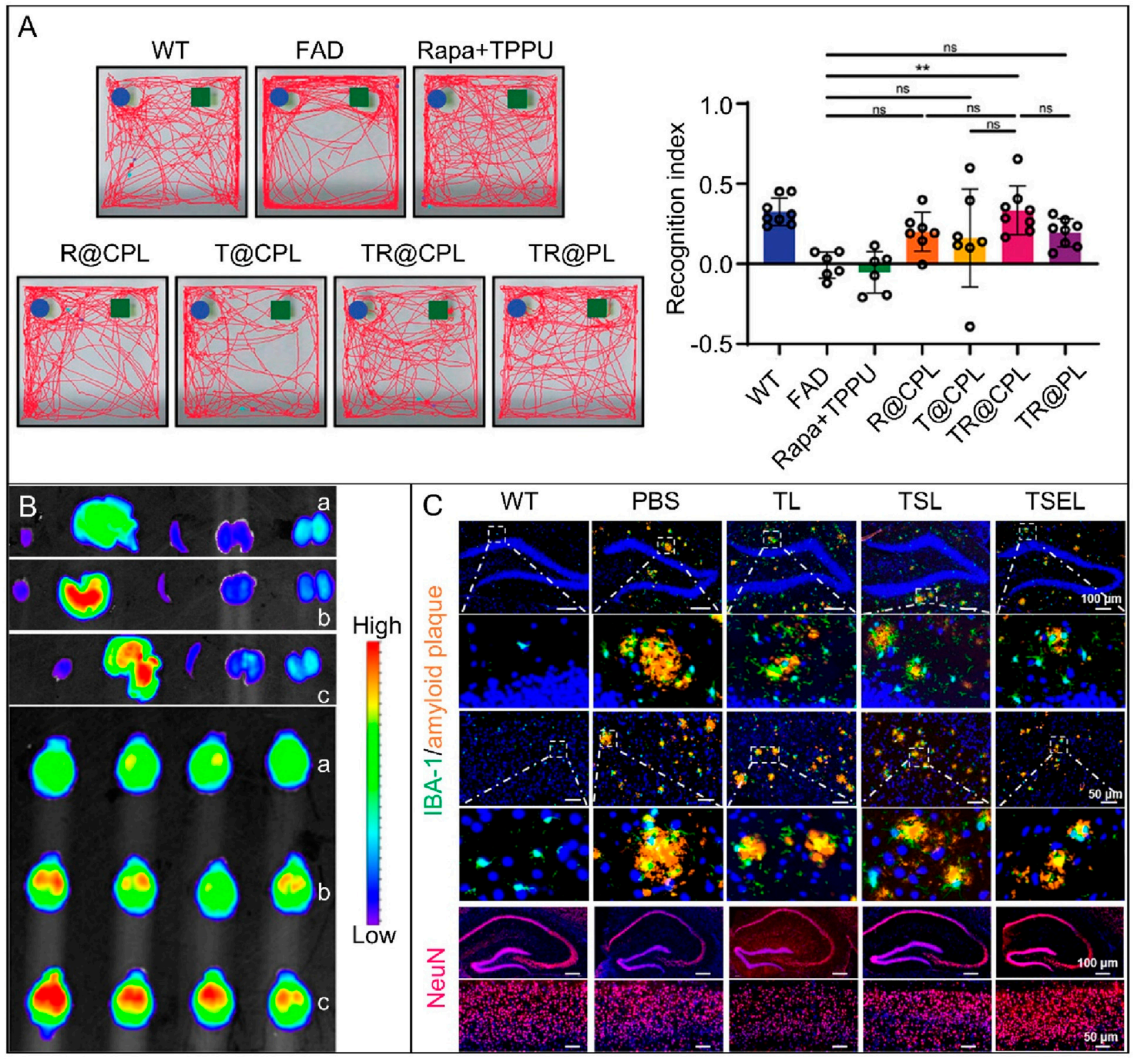
**(A)** A behavioral study on 5xFAD mice treated with different liposomes was conducted. The image shows representative trajectories of the seven groups in the NOR test and the recognition index (Lin et al., 2024). (n = 6–8, *p < 0.05, ***p < 0.001, ns *p* > 0.05). Copyright ^©^ 2024 The Authors. Advanced Science published by Wiley-VCH GmbH. **(B)** *In vitro* fluorescence imaging of the main organs (heart, liver, spleen, lung, kidney) and brain of FAD4T transgenic mice 6 h after oral administration of different fluorescent nanoparticles. (a) 28% PEG NPs, (b) 50% Man NPs, and (c) 50% Man NPs with Glu control (Lei et al., 2024). Copyright ^©^ 2024, American Chemical Society. **(C)** Immunofluorescence images of mouse hippocampus and cortex. Images show amyloid plaques (orange) and microglia (green) in the hippocampus and cortex, as well as NeuN (red) in the hippocampus of AD mice treated with PBS, TL, TSL, or TSEL. The bottom row of each image displays an enlarged view of the area indicated by the white box in the upper image (Jiang et al., 2024). Copyright ^©^ 2024, American Chemical Society.

Liposomes are biocompatible and biodegradable nanoparticles that can encapsulate a variety of drugs and release them controllably (Zhang et al., 2025). Through specific changes and modifications, liposomes can be engineered to cross the BBB and reach target areas in the brain with precision, facilitating efficient drug delivery (Zhang et al., 2019; Erel-Akbaba et al., 2025). In the Figure 3A, a novel cell membrane coating was developed by hybridizing platelets and chemokine receptor 2 cells, Multitarget therapy was achieved by loading two drugs onto liposomes with different mechanisms of action. An experimental transgenic mouse model of familial AD reduced amyloid plaque deposition, neuroinflammation, and cognitive impairment when hybrid cell membrane liposomes were loaded with drugs (Lin et al., 2024). The delivery of monoclonal antibodies into neurons is enhanced by brain-targeted liposomes (BTL), enabling intracellular and extracellular treatments for PD brains. The modified BTL was loaded with a monoclonal antibody called SynO4, which inhibits α-SYN aggregation. The findings demonstrated that 100 nm BTL traversed the human BBB model and was absorbed by primary neurons. SynO4 binds to its target neurons, reducing α-SYN aggregation and strengthening neuronal viability. *In vivo*, BTL treatment improves mouse motor function and learning ability, with a favorable safety profile (Sela et al., 2023). Combining transcranial focused ultrasound, intravesical microbubbles (MBs), and calcium phosphate lipid nanoparticles to deliver superoxide dismutase 1 (SOD1) antisense oligonucleotide to the brains of amyotrophic lateral sclerosis transgenic mice significantly enhanced delivery efficiency. Treatment resulted in decreased SOD1 expression, increased motor neurons, and a short-lived, undamaged BBB opening with reasonable tolerance (Ediriweera et al., 2025). Liposome drug carriers have been demonstrated to enhance their penetration ability across the BBB via surface modification techniques, such as PEGylation or ligand modification. The double-layer structure of the film has been shown to effectively package drugs, prolong circulation time, and improve their stability. The ability of liposomes to be absorbed by brain endothelial cells through receptor-mediated endocytosis facilitates efficient drug delivery, reduces drug distribution in non-target tissues, minimizes adverse effects, and offers an effective and safe approach to treat NDDs.

### 3.2 Polymer nanoparticles

A polymer nanoparticle as a drug carrier must be designed with the appropriate materials. These nanoparticles need to be multifunctional to ensure drug delivery to the target organs. The physical and chemical properties of nanoparticles’ affect their behavior in the body, including interactions with biological processes (Li et al., 2021; Wu et al., 2025). Therefore, an in-depth study of the relationship between nanoparticle properties and diseases is crucial for optimizing drug delivery systems performance. In general, polymer drug carriers fall into two types: natural and synthetic. Natural polymers, such as chitosan and sodium alginate, are widely used due to their excellent biocompatibility and biodegradability. Synthetic polymers, such as poly (lactic-co-glycolic acid) (PLGA) and polyethylene glycol (PEG), are favored for their adjustable physicochemical properties and controllable drug release characteristics (Katila et al., 2022). Selecting the appropriate polymer materials can enhance drug stability and delivery efficiency, reduce immune responses and side effects, and improve therapeutic effects. For the multi-target treatment of AD, an oral, brain-targeted nanoparticle containing fingolimod (FTY) is constructed with a PLGA-PEG skeleton. It is surface-modified with mannose, combined with a glucose control strategy, and its hydrophilic and electronegative properties enable penetration of the mucus barrier. The mannose ligand gives it targeting ability. Blood glucose control allows nanoparticles to bypass the BBB through GLUT 1 (Figure 3B). The FTY regulates microglia polarity, shifting them from a pro-inflammatory M1 state to an anti-inflammatory M2 state once released. This process normalizes activated astrocytes, enhances Aβ clearance, and alleviates oxidative stress and neuroinflammation (Lei et al., 2024). In particular, it demonstrates that polymer nanoparticles with tailored physicochemical properties, such as size, shape, and surface charge, can efficiently cross the BBB. By combining their multiple functions, these drugs can deliver targeted drugs and sustain release, resulting in improved efficacy and reduced side effects.

### 3.3 Exosomes

Exosomes are natural, small, cell-secreted vesicles that can pass through biological barriers. Unlike synthetic carriers, they are less likely to trigger immune responses. This makes them promising nanocarriers for therapeutic biomolecules (Kalluri and LeBleu, 2020; Rehman et al., 2023). To load proteins into exosomes, the cargo proteins need to be fused with the exosome-labeled proteins. Then, after reaching the target location, the cargo protein must be separated from the exosome marker protein to enter the recipient cell and function (Wang et al., 2025a). Almost all cells can secrete exosomes, including immune cells, nerve cells, stem cells, etc. Exosomes from different sources vary in composition and function. For instance, exosomes derived from mesenchymal stem cells have immunomodulatory and neuroprotective effects (Zhang G. et al., 2023), while those from nerve cells are closely related to neural signal transmission and synaptic function (Wu et al., 2023b). These characteristics give exosomes unique advantages in the treatment of NDDs. Exosomes can carry therapeutic substances such as small molecule drugs, nucleic acids (such as siRNA and miRNA) (Seyedaghamiri et al., 2023; Yuyama et al., 2024; Dar et al., 2021). For example, exosomes engineered to have multiple targeting capabilities (RPDA@Rb-A) were developed by combining brain microvascular endothelial cells and macrophage membranes with polydopamine nanoparticles, resveratrol, and Aβ-targeting aptamers. This combination is intended to intervene in Aβ clearance and regulate microglial dysfunction. It has demonstrated that RPDA@Rb-A degraded Aβ aggregates via local heating induced by a near-infrared laser, alleviated neurotoxicity, reduced Aβ load, and effectively inhibited microglial activation (Du et al., 2025). The ROS-responsive biomimetic exosome-liposome hybrid nanovesicle (TSEL) can modulate the function of microglia and interfere with the synthesis and metabolism of Aβ. Figure 3C shows that, compared to PBS-treated mice, TSEL-treated mice have significantly fewer Aβ plaques in the cortex and hippocampus, promoting Aβ phagocytosis by microglia. Furthermore, TSEL treatment results in the greatest increase in neuronal nuclei antigen (NeuN). These results demonstrate that TSEL can improve cognitive deficits in APP/PS1 mice by acting synergistically with two genetic drugs to regulate the phenotype of activated microglia, reduce Aβ accumulation, and prevent the re-triggering of neuroinflammation (Jiang et al., 2024). In addition, exosomes can also serve as a medium for intercellular communication, transmitting bioactive molecules and regulating the functions of diseased cells, providing new ideas for the treatment of NDDs.

### 3.4 Viral vectors

Viral vectors serve as a type of delivery system that possesses natural transmembrane transport capabilities, and they have demonstrated potentials in achieving brain-targeted drug delivery across BBB by emulating the natural infection mechanisms of viruses (He et al., 2025; Wang et al., 2025b). These vectors are able to penetrate biological barriers through receptor-mediated endocytosis on the surface of host cells. The delivery efficiency of these vectors is contingent upon the specific interactions between surface antigens and BBB endothelial cell receptors. The employment of genetic engineering technology to direct the modification of viral capsid proteins can result in a substantial enhancement of selectivity for target cells within the brain, accompanied by a concomitant reduction in off-target effects on non-target organs. For example, engineered adeno-associated virus vectors can target molecules, such as transferrin receptors, which are expressed in high quantities on the surface of brain microvascular endothelial cells. These receptors initiate receptor-mediated endocytosis and transendocytosis processes, facilitating the efficient transportation of therapeutic genes, including neuroprotective factors and enzymes, to the parenchymal cells within the brain (Huang et al., 2024a). BBB is a physiological barrier to restrict the delivery of mRNA to neurons in the brain. But extracellular vesicles containing retrovirus-like capsid mRNA produced by engineered white blood cells can enhance mRNA delivery and neuronal uptake, and cross BBB using innate adhesion molecules and recruited proteins. In a mouse model of neuroinflammation, these extracellular vesicles can help neurons take in more mRNA (Gu W. et al., 2024). However, this system continues to confront challenges related to immunogenicity, vector capacity limitations, and long-term safety concerns. Repeated administration of the substance may elicit a host immune response, leading to the clearance of the vector. The applications of viral vectors in crossing BBB have gradually advanced from basic research to preclinical validation. The combined applications of viral vectors with other delivery strategies, such as nano-vector coating to reduce immunogenicity, offering new strategies to treat neurodegenerative diseases.

### 3.5 Focused ultrasound (FUS)

FUS represents a non-invasive physical regulation method that provides an innovative approach for the reversible opening of the BBB and brain drug delivery through the synergistic effect with intravenous MBs (Rezai Ali et al., 2024; Memari et al., 2024). The mechanism could be explained by the activation of MB for ultrasonic energy (Li et al., 2023). The application of low-frequency ultrasound initiates periodic oscillations (stable cavitation) within the blood vessels, resulting in the generation of mechanical stress. The transmission of this stress to the endothelial cells of brain microvascular results in the temporary release of tight junctions (Kwak et al., 2024). It has the capacity to upregulate integrated membrane proteins, including caveolin-1 to enhance vesicle-mediated transcellular transport (Pandit et al., 2020b). And it also could regulate down P-gp to reduce drug efflux (Aryal et al., 2017). MB endure inertial cavitation under hypersonic pressures, forming shock waves and micro-jets. These phenomena temporarily compromise cell membranes and enhance the permeation of pharmaceutical agents. In addition, the local thermal effect surrounding the MB may indirectly enhance permeability by altering the characteristics of the endothelial cell membrane (Klotz et al., 2010). The combined impacts of the aforementioned mechanical and thermal mechanisms achieve local, transient and controllable permeability regulation of BBB.

Due to high spatiotemporal specificity, non-invasiveness and repeatability, FUS is frequently employed in conjunction with nano-delivery systems to enhance the efficacy of drug delivery in NDDs. Ediriweera et al. found that combining transcranial focused ultrasound and calcium phosphate lipid nanoparticles can significantly boost the delivery of SOD1 antisense oligonucleotides to the brains of transgenic mice with amyotrophic lateral sclerosis (Ediriweera et al., 2025). This approach reduces the abnormal expression level of SOD1 in the mouse brain, retains the number of motor neurons and limits the open state of BBB. The animal model tolerates the treatment well and shows no signs of neurotoxicity. The study suggests that FUS-mediated BBB opening can create a “spatiotemporal window” for nanocarriers such as liposomes and polymer nanoparticles to traverse barriers, increasing local drug targeting and reducing off-target effects caused by systemic administration. However, this technology has a limited focused area and is not very adaptable, such as the MB have a brief half-life and are unstable (Zhang et al., 2016). Thus, comprehensive understanding of ultrasound, MB, and tissue response, along with optimized regulation of cavitation effects, will enhance the safety and efficacy of the technology, facilitating its integration into brain disease treatment.

### 3.6 Intranasal delivery

The intranasal delivery of pharmaceuticals has developed as a significant approach for transcending BBB and achieving targeted brain delivery (Shen et al., 2025; Agrawal et al., 2018). This method utilizes the anatomical and structural characteristics that exist between the nasal cavity and the CNS to facilitate drug delivery to brain. The drug generally be delivered through two distinct pathways. In the intracellular pathway, drugs deposited in the olfactory epithelium can be transported along the nerves to the olfactory bulb (Sasaki et al., 2023). In the extracellular pathway, drugs enter the cerebrospinal fluid through the paracellular space of the nasal epithelial cells and subsequently diffuse into the subarachnoid space of the brain via the perineural space (Pardeshi and Belgamwar, 2013). Furthermore, drug can be absorbed through the nasal blood vessels, subsequently entering the brain via the systemic circulation. This process has the potential to mitigate the systemic toxic and side effects which are associated with systemic administration, circumvent the initial hepatic metabolism, diminish the accumulation of drugs in non-target tissues, and concurrently augment the targeted enrichment level in the brain (Huang et al., 2024b).

In intranasal drug delivery systems, nanocarriers such as polymers, liposomes, and micelles have been utilized extensively (Wang et al., 2019). Polymer nanoparticles have garnered significant attention due to their inherent safety, their ability to encapsulate drugs with high efficiency, and their versatile structural tunability (Jia et al., 2025a). The presence of mucosal adhesion properties can prolong retention time in nasal cavity to reduce mucociliary clearance. A system based on exosome has been demonstrated to facilitate the delivery of neuroprotective peptides and nucleic acids to brain lesion areas, which could be contributed to its natural biocompatibility and barrier penetration ability. In models of AD, it has been observed to modulate microglia phenotype and facilitate Aβ clearance (Xu et al., 2025). Notably, the delivery efficiency of intranasal formulations is determined by the physicochemical properties of drugs and the characteristics of nanocarriers. The passive diffusion and absorption of lipophilic small molecules by the olfactory epithelium is well-documented, while the necessity of modifying nanocarriers for epithelial barrier breakthrough is well-established for drugs. The nanoscale size and negative charge of lipid nanoparticles facilitate their transportation through olfaction and the trigeminal nerve (Jia et al., 2025b). Viscosity-control strategies enhance naso-brain delivery efficacy (Yue et al., 2025). Although intranasal administration has advantages, its clinical application still faces challenges. The nasal cavity’s enzymes and mucociliary clearance affect drug stability, and the dosage is limited. Cationic carriers enhance mucosal adhesion, but pose risks of systemic and local toxicity. Exploration of how the intranasal delivery system interacts with the nasal mucosa and neural pathways will improve the therapeutic effect of NDDs.

## 4 Strategies for drug delivery systems to cross the BBB

### 4.1 Passive targeting strategy

**FIGURE 4:**
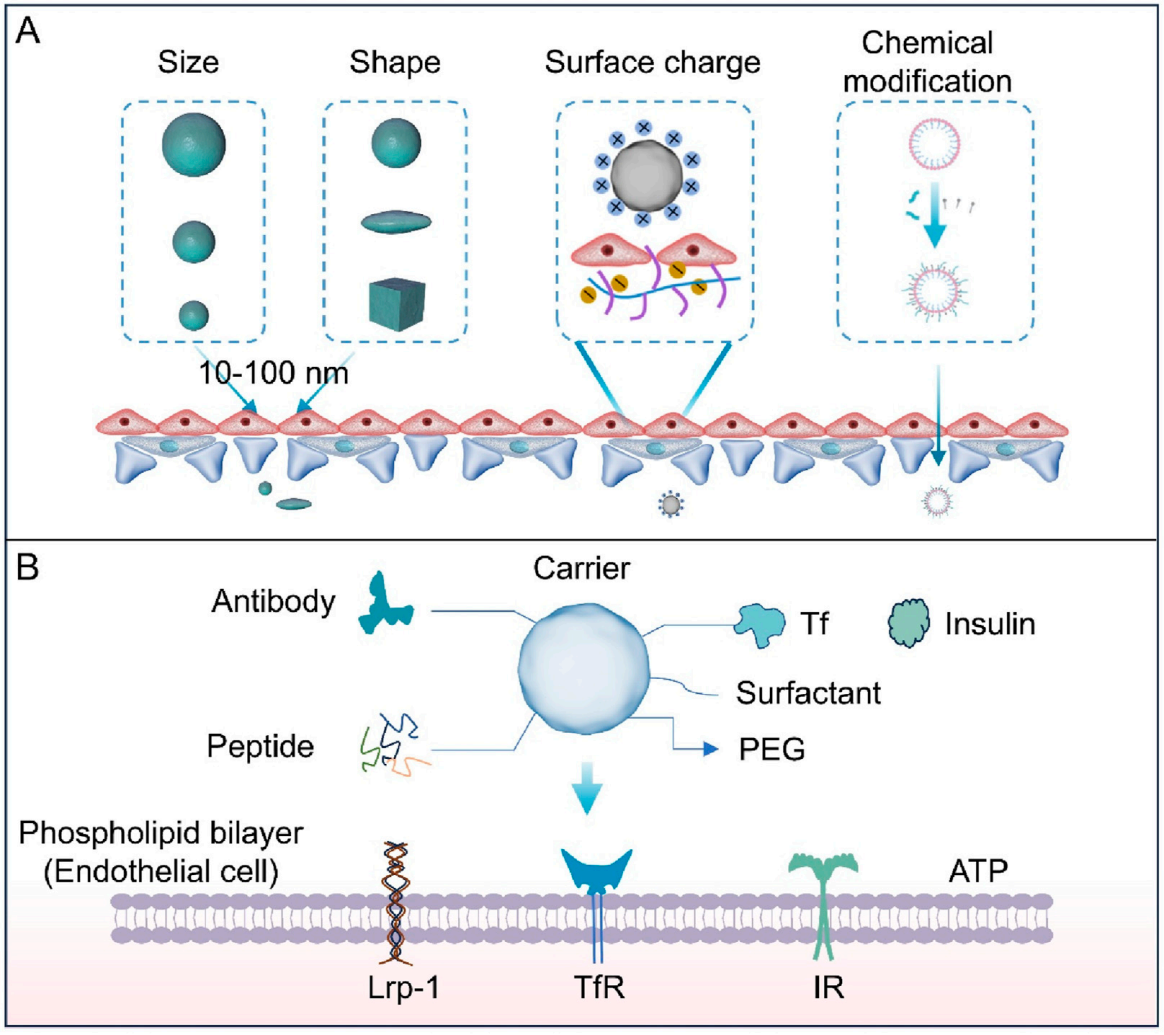
**(A)** The size, shape, chemical modification, and surface charge of nanoparticles all affect their ability to penetrate the BBB. Particles with small size, rod-shaped structures, lipophilic properties, or positive surface charge are conducive to crossing endothelial junctions. **(B)** Ligands include antibodies, transferrin, insulin, and peptides. Chemical groups include polyethylene glycol (PEG) and surfactants. These surface-functionalized groups promote the entry of carriers through the BBB and their absorption by the brain. Carriers modified with ligands can be recognized by cell surface receptors such as transferrin receptor (TfR), low-density lipoprotein receptor-related protein 1 (LRP-1), and insulin receptor (IR). This recognition promotes transcellular transport of the carriers, while chemical groups (e.g., PEG) enhance stability, prolong circulation time, or reduce non-specific interactions.

Passive strategies for BBB penetration constitute a form of non-specific transport. This category does not rely on specific targeting molecules, instead primarily leveraging the physiological characteristics or pathological states of the BBB, or achieve cross-barrier transport by optimizing the physicochemical properties of the carrier (Table 1). During the pathological process of NDDs such as AD, PD, and MS, the tight junctions of the BBB gradually loosen due to chronic inflammation, oxidative stress and other factors, and the permeability abnormally increases. Drugs or nanocapsules can passively diffuse into the brain parenchyma through open paracellular pathways. For instance, in the acute phase of MS, inflammatory factors (such as tumor necrosis factor-α and interleukin-1β) can disrupt the tight junction proteins between endothelial cells (such as claudin-5 and occludin), causing the local opening of the BBB. Anti-inflammatory drugs can thereby non-specifically penetrate into demyelinating lesions (Zierfuss et al., 2024). In addition, by optimizing the physical properties of drug delivery nanoparticles (Figure 4A), including size, shape and surface charge, nanoparticles can more effectively cross the BBB through passive transport mechanisms, thereby improving the distribution and efficacy of drugs in the brain (Nance et al., 2012; Wu et al., 2025; Wen et al., 2023). Smaller nanoparticles (typically less than 100 nm) are more likely to pass through the BBB because they are more easily taken up by brain endothelial cells (Cruz et al., 2016). In addition, nanoparticle shape also affects their behavior in the BBB. Spherical nanoparticles usually have superior biocompatibility and lower immunogenicity, while rod-shaped or flaky nanoparticles may have higher cellular uptake efficiency (Banerjee et al., 2016; Nowak et al., 2020). Nanoparticle surface charge is equally relevant. There is a higher rate of cellular uptake of positively charged nanoparticles, but they may cause a stronger immune response as well. Negatively charged nanoparticles, on the other hand, have better stability and lower immunogenicity (Chen et al., 2024; Wang et al., 2025c). Nano-carriers with surface-modified neutral or weak anionic groups (such as nanoparticles coated with hyaluronic acid) can reduce non-specific binding to blood components and enter the brain parenchyma through adsorption-mediated endocytosis. They are particularly effective in NDDs with mild BBB damage, such as PD.

**TABLE 1:** Typical strategies for drug delivery across the BBB.

Strategy Type		Mechanism	Advantages	Limitations	Materials/techniques	Specificities	Experimental Models	References
Passive transport	Physicochemical-dependent	Dependent on BBB disruption (e.g., pathological permeability) or carrier properties (small size, lipophilicity) for passive diffusion.	Simple preparation, low cost; suitable for BBB-damaged models.	Limited to pathological conditions; high off-target toxicity.	Unmodified nanoparticles (<100 nm), liposomes (relying on BBB leakage), lipophilic small molecules.	—	Glioblastoma mice, stroke models	Cruz et al. (2016), Nowak et al. (2020), Chen et al. (2024)
Active targeting	Ligand-mediated targeting	Specific binding of ligands to high-expression BBB receptors, triggering receptor-mediated endocytosis.	Independent of BBB integrity; high specificity, reduced off-target distribution.	Dependent on high receptor expression; potential immune responses.	Antibody-based: AAV capsid targeting hTfR	hTfR, LRP-1, LfR.	AD model mice, MS model mice, monkeys	Huang et al. (2024a)
					Fc fragment BBB transport vehicle (hTfR)			Kariolis et al. (2020)
					Peptide-based: Angiopep-2 modified nanoparticles (LRP-1).			Katila et al. (2022)
					Small molecule-based: Lactoferrin-modified PLGA nanoparticles (LfR)			Zhao et al. (2024)
Physicochemical optimization (Auxiliary)	—	Enhances stability/circulation (e.g., PEGylation) or endosome escape (e.g., pH-sensitive materials).	Improves delivery efficiency of passive/active strategies.	Excessive modification may affect ligand-receptor binding.	PEGylated liposomes	—	AD model	Gu et al. (2024b)
					porous coordination network-224 nanoparticles		Glioblastoma	Cao et al. (2022)

AAV: adeno-associated virus, hTfR: the human transferrin receptor, LRP-1: low-density lipoprotein receptor-related protein 1, LfR: lactoferrin receptor.

Further optimization of physicochemical properties of drug carriers or molecules can enhance their penetration of the BBB through surface or chemical modification. Among them, surface modification can increase contact probability with the BBB by prolonging the circulation time, improving penetration efficiency through passive diffusion. PEGylation reduces their clearance rate in the blood, prolongs circulation time, and reduces non-specific adsorption (Chen et al., 2024; Gu Z. et al., 2024). For example, to facilitate the delivery of the drug to the brain, ATX was encapsulated with liposomes and then conjugated with PEG to produce liposome nanoparticles (PEG-ATX@NPs). PEG-ATX@NPs have been demonstrated to reduce Aβ neurotoxicity by degrading FA *in vitro* and reducing FA-induced Aβ assembly. Chemical modification, by contrast, focuses on enhancing transmembrane properties. Given the high lipophilicity of the BBB’s endothelial cells, chemical modification primarily enhances passive diffusion by improving the lipid solubility of carriers or drugs (Zhou et al., 2018; Hajal et al., 2021). Therefore, researchers have explored a variety of lipid-based drug delivery systems to enhance BBB permeability while considering the biodegradability of the materials. This is crucial for controlling the rate of drug release from nanoparticles and the pharmacokinetics of drugs (Wang et al., 2025c). Fatty acid esterification augments transmembrane diffusion by leveraging the BBB’s lipid bilayer structure, thereby improving brain permeability (Xue et al., 2025; Wang Y. et al., 2025). However, passive strategies are primarily applicable when the BBB is pathologically damaged, and their non-specific nature may cause systemic vascular permeability to increase, leading to toxic side effects in peripheral tissues.

### 4.2 Active targeting strategy

The active targeting strategy does not rely on the inherent transport mechanisms of the BBB, but instead actively regulates or enhances specific transport pathways through targeted design, thereby improving the efficiency and specificity of drug delivery to the brain (Table 1). Its core lies in leveraging highly expressed specific receptors on BBB endothelial cells to trigger energy-dependent active transport by ligand-receptor recognition, thus overcoming the constraints of an intact BBB (Figure 4B).

#### 4.2.1 Ligand-mediated targeted modification

Ligand-mediated targeting relies on specific interactions between ligands and receptors on the BBB, triggering receptor-mediated endocytosis or transport to enhance brain delivery. This strategy leverages high-expression receptors on BBB endothelial cells to improve targeting efficiency and reduce non-specific distribution (Li and Kataoka, 2021). Common ligands include antibodies, peptides, and small molecules, with key targeting receptors such as transferrin receptor (TfR), lactoferrin receptor (LfR) and LRP-1 (Huang et al., 2024a; Cao et al., 2022; Sagare et al., 2012) As a type of immunoglobulin that can specifically bind to antigens/receptors, antibody ligands, with their extremely high affinity and targeting specificity, have become the most commonly used method in the active targeting of BBB. TfR and insulin receptor, which are highly expressed in BBB endothelial cells, are typical targets. Tf antibodies or transferrin-modified carriers (e.g., liposomes, nanoparticles) specifically bind to TfR, enabling receptor-mediated endocytosis to cross the BBB (Song M.-s. et al., 2025; Kariolis et al., 2020). A microglial exosome-Tf-liposome hybrid carrier delivers berberine and palmatine to the brain. Tf-modification facilitates BBB penetration, while berberine inhibits β-secretase and palmatine regulates NF-κB signaling, improving cognitive dysfunction in AD models (Zhou et al., 2025). Peptide ligands composed of short-chain amino acids have advantages such as small molecular weight (<5 kDa), high biocompatibility, and ease of chemical synthesis/modification, making them particularly suitable for penetrating the tight structure of BBB endothelial cells. For instance, peptides targeting LRP-1 (e.g., Angiopep-2) mimic endogenous peptide sequences to bind specifically to receptors, facilitating receptor-mediated endocytosis across the BBB (Zhao et al., 2024; Martikainen et al., 2024; Yoshioka et al., 2025).

Small molecule ligands form specific binding with BBB receptors through chemical structures, featuring high stability, easy batch synthesis and low cost. Unlike antibody-based targeting, they are particularly suitable for chemical coupling with nanocarriers. Lactoferrin (Lf), a natural ligand of LfR, enables modified nanoparticles to increase the intracranial concentration of anti-inflammatory drugs in the MS model. Moreover, due to the high specificity of Lf and LfR, its penetration into the normal BBB area is extremely low (Zierfuss et al., 2024). A lipid nanocomposite modified with apolipoprotein A-I, its mimetic peptide 4F, and angioendothelin-2 crosses the BBB, targets microglia, eliminates amyloid-β, and inhibits tau phosphorylation, which significantly improves AD pathology (Han et al., 2022).

#### 4.2.2 Chemical coupling and functional optimization of ligands

The targeting effect of ligands not only depends on their type, but also on the coupling mode with the carrier and functional optimization (Wang C. et al., 2024). Fixing ligands to the surface of carriers through chemical coupling is a key technology to enhance targeting efficiency. The core lies in strengthening the binding ability of carriers to BBB endothelial cells while ensuring the activity of ligands. The selection of coupling methods, the retention of ligand activity, and the coordinated optimization of carrier functions directly affect the final effect of active targeting. The chemical coupling of ligands and carriers needs to take into account both the stability of the bond and the biological activity of the ligands. The appropriate method should be selected based on the type of ligand (such as antibodies, peptides) and the characteristics of the carrier material (Wu et al., 2019). The use of flexible connection tools such as PEG can reduce steric hindrance. For instance, PEG-coupled Tf enhanced liposomes’ ability to cross the BBB, which to some extent could be attributed to the reduction of steric hindrance by PEG. This improved interaction with the highly expressed transferrin receptor on the blood-brain barrier in PD (Sela et al., 2023). Although these strategies enhance the delivery of drugs to the brain through ligand-receptor interactions, they have limitations, such as dependence on high receptor expression and the potential for immune responses or receptor downregulation. Existing solutions include developing multi-ligand collaborative targeting to enhance binding efficiency and using low immunogenic ligands to reduce immune responses. Other solutions involve allosteric targeting and stimulus-responsive release techniques.

## 5 Clinical translational analysis

Preclinical studies use rodent models, such as mice and rats, and non-human primates, including rhesus monkeys and Beagle dogs, to assess the penetration efficiency of BBB, the targeting ability of vectors, and the efficacy of these vectors (Deo et al., 2013). In the course of the clinical trial stage, a multitude of vectors have been investigated. Doxorubicin liposomes were previously included in the phase I clinical trial of glioma (Barenholz, 2012). However, the trial was terminated due to the tumour suppression rate falling short of the stipulated endpoint. The preliminary phase of clinical trials has demonstrated the safety of intranasal administration of allogenic human adipose mesenchymal stromal cells-derived exosome in the treatment of AD. The clinical trial (NCT04388982) for the treatment of AD with exosome nasal spray has enrolled nine patients, completed administration and follow-up, and no serious adverse events occurred (Xinyi et al., 2023). Nevertheless, it is imperative to acknowledge that transferrin-coated liposomes are currently in the preclinical phase of research.

Despite the advances made by the aforementioned carriers in the realm of clinical research, the lack of clinical approval for nanocarriers can be attributed to several fundamental issues. The divergent structures of the BBB in rodents and humans complicate the extrapolation of preclinical data to humans (Hoshi et al., 2013). It has been demonstrated that both large-scale production and stability are susceptible to disruption due to bottlenecks. The fluidity of liposome membranes and the composition of exosome surface proteins are affected by multiple factors and do not meet the standards for clinical batch production. However, the dearth of long-term safety data and the elusive nature of the metabolic pathways of nanoparticles in the brain represent significant obstacles (Zhao et al., 2018). The potential neurotoxic risks associated with this process have not yet been ruled out. The regulatory and evaluation system is deficient, and there is an absence of unified clinical endpoint indicators (Younis et al., 2022). In summary, the clinical transformation of delivery systems must address species differences, production process limitations, and regulatory barriers. Cross-scale model validation and the establishment of a standardized evaluation system are necessary to facilitate the transition of these models from laboratory to clinical settings.

## 6 Conclusion and prospect

At present, various drug delivery systems such as liposomes, nanoparticles, exosomes and viral vectors have made certain progress in penetrating the BBB for NDDs through strategies such as surface modification and structural modification. The modified vectors can cross the BBB by means of mechanisms such as receptor-mediated endocytosis and adsorption-mediated endocytosis, achieving precise drug delivery to the lesion areas of the brain and demonstrating potential therapeutic effects in the treatment and research of diseases such as AD and PD. However, as previously noted, these systems face persistent challenges. Such as the poor stability of liposomes and limited drug loading capacity; The long-term safety and *in vivo* metabolic processes of nanoparticles remain unclear. The preparation process for exosomes is complex and costly. Furthermore, the pathological mechanisms of different NDDs vary significantly, and a single delivery strategy is difficult to meet the therapeutic needs of all diseases. In the future, further in-depth research is needed on the physiological characteristics and regulatory mechanisms of the BBB to develop safe, and promote targeted efficiency drug delivery systems. At the same time, by integrating multi-disciplinary technologies, an organic combination of drug delivery and disease-specific treatment can be achieved, promoting substantive breakthroughs in neurodegenerative disease treatment.
